# Assessment of effectiveness and safety of repeat administration of proinflammatory primed allogeneic mesenchymal stem cells in an equine model of chemically induced osteoarthritis

**DOI:** 10.1186/s12917-018-1556-3

**Published:** 2018-08-17

**Authors:** Laura Barrachina, Ana Rosa Remacha, Antonio Romero, Arantza Vitoria, Jorge Albareda, Marta Prades, Mercedes Roca, Pilar Zaragoza, Francisco José Vázquez, Clementina Rodellar

**Affiliations:** 1 Laboratorio de Genética Bioquímica LAGENBIO - Instituto Agroalimentario de Aragón IA2 - Instituto de Investigación Sanitaria de Aragón IIS, Universidad de Zaragoza, C/Miguel Servet, 177, 50013 Zaragoza, Spain; 2 Servicio de Cirugía y Medicina Equina, Hospital Veterinario, Universidad de Zaragoza, C/Miguel Servet, 177, 50013 Zaragoza, Spain; 3 Servicio de Cirugía Ortopédica y Traumatología, Hospital Clínico Universitario Lozano Blesa, Zaragoza. Avda. San Juan Bosco, 15, 50009 Zaragoza, Spain; 4 Departament de Medicina i Cirugia Animal, Universidad Autónoma de Barcelona, Edifici H, UAB, 08193 Bellaterra, Barcelona, Spain; 5 Clínica Doctora Roca Diagnóstico Médico, Carrera del Sábado 4, local (Edificio Europa), 50006 Zaragoza, Spain

**Keywords:** Cartilage, Horse, Joint pathology, Immunogenicity, Immunomodulation, Synovial fluid

## Abstract

**Background:**

This study aimed at assessing the effectiveness and safety of repeated administrations of allogeneic bone marrow-derived mesenchymal stem cells (BM-MSCs) primed with tumor necrosis factor (TNF)-α and interferon-γ in an equine model of chemically-induced osteoarthritis. Arthritis was induced in both radio-carpal (RC)-joints by amphotericin-B in 18 ponies, divided into three groups depending on the treatment injected: MSC-naïve (*n* = 7), MSC-primed (*n* = 7) and control (*n* = 4). The study consisted of two phases and used one RC-joint of each animal in each phase, with four months time-lapse, in order to assess two end-points. Clinical, synovial, radiological and ultrasonographic follow-up was performed. At six months, animals were euthanized and both carpi were assessed by magnetic resonance imaging (MRI), gross anatomy, histopathology, histochemistry and gene expression.

**Results:**

Clinical and synovial inflammatory signs were quicker reduced in MSC-treated groups and repeated allogeneic administration did not produce adverse reactions, but MSC-primed group showed slight and transient local inflammation after second injection. Radiology and MRI did not show significant differences between treated and control groups, whereas ultrasonography suggested reduced synovial effusion in MSC-treated groups. Both MSC-treated groups showed enhanced cartilage gross appearance at two compared to six months (MSC-naïve, *p* < 0.05). Cartilage histopathology did not reveal differences but histochemistry suggested delayed progression of proteoglycan loss in MSC-treated groups. Synovium histopathology indicated decreased inflammation (*p* < 0.01) in MSC-primed and MSC-naïve at two and six months, respectively. At two months, cartilage from MSC-primed group significantly (*p* < 0.05) upregulated collagen type II (COL2A1) and transforming growth factor (TGF)-β1 and downregulated cyclooxygenase-2 and interleukin (IL)-1β. At six months, MSC-treatments significantly downregulated TNFα (*p <* 0.05), plus MSC-primed upregulated (*p <* 0.05) COL2A1, aggrecan, cartilage oligomeric protein, tissue inhibitor of metalloproteinases-2 and TGF-β1. In synovium, both MSC-treatments decreased (*p* < 0.01) matrix metalloproteinase-13 expression at two months and MSC-primed also downregulated TNFα (*p <* 0.05) and IL-1β (*p <* 0.01).

**Conclusions:**

Both MSC-treatments provided beneficial effects, mostly observed at short-term. Despite no huge differences between MSC-treatments, the findings suggested enhanced anti-inflammatory and regulatory potential of MSC-primed. While further research is needed to better understand these effects and clarify immunogenicity implications, these findings contribute to enlarge the knowledge about MSC therapeutics and how they could be influenced.

**Electronic supplementary material:**

The online version of this article (10.1186/s12917-018-1556-3) contains supplementary material, which is available to authorized users.

## Background

The lack of effective treatments for joint pathologies such as osteoarthritis (OA) has risen interest in therapies based on mesenchymal stem cells (MSCs) [1]. Provided that their engraftment in the cartilage appears to be low [2, 3], MSC benefit is being mainly attributed to their paracrine mechanisms including trophic and immunomodulatory properties. Therefore, it has been a shift towards using MSCs to regulate joint inflammation rather than to replace damaged cartilage [4].

Since the main effect of MSCs in OA appears to be related to their immunomodulatory and anti-inflammatory properties [5], the choice of the lesion model to study these abilities is an important aspect. Enhanced therapeutic benefit of MSCs has been shown in vitro and in vivo when a remarkable level of proinflammatory mediators is present [6, 7], so highly inflammatory OA models could be suitable for studying the regulatory role of MSCs [8]. Conditions producing acute joint inflammation can lead to a prolonged inflammatory situation and subsequent degradation of the articular cartilage, which eventually produces degenerative changes in the joint [9, 10]. Chemically induced-OA models are used to mimic this process in different species, including the horse, since they are characterized by a significant initial inflammatory component. Amphotericin-B intra-articular (IA) injection has been used to induce acute arthritis in the horse which can lead to degenerative changes in the cartilage [11–13]. The inflammation generated includes elevated matrix metalloproteinases (MMP) activity during several weeks, to an extent capable of promoting degradation of the extracellular matrix (ECM) of the articular cartilage [12]. Furthermore, MMP dysregulation plays a key role in OA progression since the cartilage ECM disruption is mainly perpetrated by enzymatic degradation [14]. Therefore, inflammation generated in this model eventually results in erosive lesions of the articular cartilage related to OA [11, 12].

Provided that inflammation plays a key role in OA pathophysiology by promoting cartilage breakdown, an optimal therapeutic approach should try to both limit inflammation and stimulate regeneration of articular structures [15]. Regulatory abilities elicited by MSCs in the inflammatory and catabolic environment of the diseased joint seem to be a multifactorial process, involving both direct cell-to-cell contact and paracrine signaling through different molecules governing the regulation of immune and inflammatory cells [3]. Mesenchymal stem cells may elicit immune regulatory effects in basal conditions, but the induction of their full immunomodulatory function requires activation by inflammatory signals [16, 17]. This fact is closely related with the aforementioned suitability of inflammatory OA models. In fact, improved MSC outcome has been shown in mice when using models characterized by high inflammatory component [6] rather than when using destabilization models [18]. Similarly, enhanced outcome of autologous MSCs has been observed in the equine amphotericin-B model [11] compared to the post-traumatic OA model [19], which has been proposed to be due to higher severity of the amphotericin-B model [20]. However, inflammatory environment encountered by MSCs in the site of injury may not be sufficient for full MSC activation [21], so in vitro MSC priming with pro-inflammatory molecules prior to their administration could be a suitable approach to enhance their therapeutic benefit [16]. Actually, tumor necrosis factor (TNF)α-priming increased in vivo MSC benefit in Achilles tendon injury rat model [22] and interferon (IFN)γ-stimulated equine MSCs showed enhanced effects in a murine OA model, supporting the potential of this strategy [8]. Furthermore, the enhanced immune suppressive properties of primed MSCs might help the allogeneic ones to evade the receptor immune system [23]. On the other hand, MSC inflammatory priming may also induce or increase the expression of major histocompatibility complex (MHC) molecules [24], with potential implications for their allogeneic use. Nevertheless, allogeneic MSCs primed with IFNγ displayed therapeutic benefit in different pathology models [23], so further investigation is needed to clarify both effectiveness and safety of primed allogeneic MSCs because of their potential benefits.

Allogeneic therapy, among other advantages, enables earlier MSC administration [25]. Even though the optimal moment for MSC treatment has yet to be elucidated, enhanced outcome has been reported in an equine model when MSCs were earlier administered after amphotericin-B arthritis induction. In that study, improvement begun at two months but was not consistently maintained at six months [11]. Thus, Mokbel and colleagues proposed that enhanced benefit could have been reached by using repeated MSC administrations [11], similarly to that reported in a porcine model of meniscal injury [26]. Repeated IA injections of allogeneic MSCs pooled from several donors are clinically safe in the equine healthy joint [27], as well as single IA administration of allogeneic MSCs into the equine pathologic joints [28, 29]. However, repeated allogeneic MSC administration in equine pathologic joints has not yet been reported, neither the effects of inflammatory primed MSCs in the horse.

Equine bone marrow derived MSCs (BM-MSCs) primed with 5 ng/ml of TNFα and IFNγ during 12 h induced their immunomodulatory profile while preserving cell viability and differentiation ability, with moderate increase of MHC-II expression [17], so these stimulatory conditions could be suitable to assess the effects of primed cells in vivo. Therefore, the aim of this study was to assess the use of repeated injections of allogeneic donor-pooled BM-MSCs, naïves (unstimulated) or primed with TNFα and IFNγ, in an equine model of chemically induced-OA to evaluate the safety and effectiveness of this strategy. We hypothesized that the induction of the immune regulatory profile of equine BM-MSCs would have a positive impact on their therapeutic effects in an equine joint pathology model.

## Methods

### Study design

The study design consisted of two phases and involved 18 animals divided into three groups (control, *n* = 4; MSC-naïve, *n* = 7; MSC-primed, *n* = 7), whose 36 radio-carpal (RC)-joints were used: 18 RC-joints (one of each animal) for phase 1 and the contralateral 18 RC-joints in the phase 2. Smaller size of the control group was selected to enlarge treatment groups, based on previously reported reliability of this design [30]. In the phase 1, acute arthritis was induced in one RC-joint of each animal (*n* = 18) through IA injection of amphotericin-B. Radio-carpal joints received two injections (Weeks 2 and 5 post-lesion induction) of corresponding substance: allogeneic pooled BM-MSCs unstimulated (MSC-naïve group, *n* = 7 RC-joints), or primed by TNFα and IFNγ (MSC-primed group, *n* = 7 RC-joints), or Lactate’s Ringer Solution (LRS) (control group, *n* = 4 RC-joints). Clinical, synovial, ultrasonographic and radiologic assessments were performed weekly until two months post-induction. The phase 2 started at four months, after additional two months interval. Lesions were induced in the contralateral RC-joint of each animal and they were subjected to the same corresponding procedure including repeated injection of the same substances and follow-up until two months. Then, at six months, animals were euthanized and magnetic resonance imaging (MRI), gross anatomy, histopathology, histochemistry and gene expression of cartilage and synovium were assessed. Schematic representation of the study design is provided in Additional file 1.

This two-phases study design aimed at evaluating two different end-points (two months for phase 2 RC-joints and six months for phase 1 RC-joints) without duplicating the number of animals, since the migration of IA injected MSCs from one joint to another appears to be unlikely [31, 32] and both joints of each animal are often used in studies involving large animal models which induce the lesions either at the same time [11, 33–35] or separately at different times [36].

### Animals

Eighteen animals (Shetland ponies, geldings, 3–7 years, 100–165 kg) were selected based on homogeneous characteristics (breed, sex, age, weight) and included in the study after ensuring good health status and lack of RC-joint abnormalities. Animals were acquired from a local equine marketplace. All animals were determined to be in good health based on clinical and hematological exams and absence of RC-joint pathology was assessed by clinical (static and dynamic exams, including range of motion and flexion test), synovial and radiological exams. The animals were randomly divided into three groups arranged as described above. Animals in the control group served as donors of allogeneic BM-MSCs. All procedures were carried out under Project License (PI 31/11) approved by the Ethic Committee for Animal Experiments from the University of Zaragoza. The care and use of animals were performed in accordance with the Spanish Policy for Animal Protection RD53/2013, which meets the European Union Directive 2010/63 on the protection of animals used for scientific purposes.

### Equine BM-MSCs isolation, characterization and culture

Animals from control group (*n* = 4) were sedated with 0.04 mg/kg IV romifidine (Sedivet, Boehringer-Ingelheim) and 0.02mg/kg IV butorphanol (Torbugesic, Pfizer) and 20–30 mL of bone marrow were collected from sternum in heparinized syringes with a Jamshidi needle. Procedures for MSC isolation, characterization and culture were performed as previously described [17]. Briefly, nucleated cells were harvested by density gradient centrifugation (Lymphoprep, Atom) and cultured in basal medium (low glucose Dulbecco’s Modified Eagle’s Medium [DMEM] with 10% fetal bovine serum [FBS], 2 mM L-glutamine, 0.1 mg/mL streptomycin and 100 U/mL penicillin [all from Sigma-Aldrich]) at 37 °C, 5% CO_2_. At passage 3, cells were characterized by flow cytometry and real time quantitative polymerase chain reaction (RT-qPCR) for surface markers expression and by tri-lineage differentiation potential according to methodology previously described [37]. Details about characterization of equine BM-MSCs can be found in Additional file 2. Subsequently, BM-MSCs were frozen in medium FBS with 10% dimethyl-sulfoxide (DMSO) (Sigma-Aldrich) (− 80 °C).

### Acute arthritis induction

Animals were sedated as described above and 25 mg of amphotericin-B (Sigma-Aldrich) were IA injected through aseptic arthrocentesis in one randomly assigned RC-joint (phase 1) [11, 38]. The same procedure was repeated four months later in contralateral RC-joints (phase 2). Animals did not receive any anti-inflammatory drug that could have interfered with the development of the arthritis. Analgesia was provided as previously described [38]: 0.1 mg/kg IV butorphanol were administered every 8 h during 3 days and additional dose was provided if animals presented heart rate (HR) > 60, respiratory rate (RR) > 48 or rectal temperature > 38.8 °C within the 8 h interval. If insufficient, perineural block of the median, radial and ulnar nerves would be performed with bupivacaine 0.5% (Braun). Animals were located in boxes during 15 days post-induction and subsequently placed in ample paddocks allowing free movement.

### Preparation of MSC-naïve, MSC-primed and control injections

Cryopreserved BM-MSCs (passage 3) were thawed seven days before each administration and cultured in basal medium described above. Medium of BM-MSCs destined to the MSC-primed group was replaced by medium containing 5 ng/mL of TNFα and IFNγ (R&D Systems) at 12 h before the administration [6]. BM-MSCs from the four donors were proportionally mixed and washed three times with PBS (Gibco) and three times with LRS (Braun). Subsequently, 10·10^6^ (containing approximately 2.5·10^6^ cells/donor) of naïve or primed BM-MSCs were suspended in 2 mL LRS and IA administered to corresponding animals. Ponies from control group received the same volume of LRS. All injections were performed blindly for the substance administered by wrapping the syringe with aluminum foil.

The effects of the cytokine priming on equine BM-MSC immunomodulatory and immunogenic profiles, as well as on their viability and differentiation potential, were already described by our group, as those were the same cells than the used in the present study. Briefly, MSC viability was well preserved after proinflammatory priming and under culture with inflammatory synovial fluid (SF) [17, 39], as shown by appropriate proliferation, absence of changes in the BAX/BCL-2 ratio and lack of DNA damage. Furthermore, immunomodulatory profile was upregulated in MSC-primed compared to MSC-naïve. On the other hand, the expression of MHC-II increased in MSC-primed, but MHC-I expression was not induced [17].

### Clinical assessment

General health status, HR, RR and rectal temperature were recorded every 8 h throughout 15 days post-lesion induction. Lameness scores were assigned [40] and joint inflammation was monitored by determining the carpal circumference and the local heat with a non-contact infrared laser thermometer (Thermolab) [27]. This complete clinical exam was performed before inducing the lesion (Time 0) and afterwards weekly until the Week 6, at two months (phase 1 and 2 carpi, *n* = 36), and at four and six months (only phase 1 carpi, *n* = 18). Additionally, clinical exam was performed 24 h after every injection.

### Synovial fluid assessment

Synovial fluid (0.5-1 mL) was collected in EDTA at Time 0 and afterwards weekly from Weeks 2 to 6, at two months (phase 1 and 2 carpi, *n* = 36) and at 6 months (only phase 1 carpi, *n* = 18). Total protein (TP) was determined by refractometry (Spectrum Technologies), total white cells count (tWCC) was recorded with a hemocytometer chamber and Diff-Quick (PreAnalityX) stained cytological preparations were examined to report the percentage of neutrophils, lymphocytes and macrophages (differential WCC, dWCC) [27].

### Haptoglobin determination in serum and SF

At the same time-points as SF collection, 5 mL of blood were harvested (*n* = 18 animals). Both SF and blood were centrifuged at 3000 g/15 min for obtaining SF supernatant and serum, which were stored at − 80 °C. Haptoglobin was determined following the methodology described [38]. Briefly, single radial immunodiffusion was carried out in 1% agarose gel containing rabbit polyclonal antisera anti-haptoglobin. Concentrations were obtained by extrapolation from a standard curve of known concentrations of commercial equine haptoglobin (Mybiosources).

### Ultrasonography and radiology

Ultrasonography was performed at the same time-points as SF collection. Radiographs were taken on Time 0, Week 2, and at two (phase 1 and 2 carpi, *n* = 36), four and six months (only phase 1 carpi, *n* = 18). Ultrasound images were taken using a Zonare Z-One machine using a linear transducer (5–14 MHz, Zonare, Inc.) that was transversally placed on the dorsal aspect of the carpus, slightly lateral, at the level of the RC-joint to assess soft tissues surrounding the joint, joint capsule, joint effusion and the articular surface of radiocarpal and intermediate carpal bones that was ultrasonographically accessible [41]. Radiographs were taken using an X-ray generator Orange 8016HF (M&I Globaltech) and 24 × 30 cm plates FCR FujiIPCassette-CC using 60 kv, 5 mAs and 60 cm focal distance, and processed using a FCR-Prima CR-IR391RU computerized radiology system (all from Fujifilm). Four of the authors (AV, JA, MP, FJV) blindly assessed the radiographs and assigned a score to each one by consensus agreement following an ordinal scale (Additional file 3.A, adapted from [11]).

### Magnetic resonance imaging

Six months since the start of the study, ponies were sedated and subsequently euthanized with 200 mg/kg IV sodium pentobarbital (Euthasol, Esteve). All the carpi (phase 1, *n* = 18; phase 2, *n* = 18) were subjected to MRI by using an AIRIS II-1 0.3 T MRI unit (Hitachi) and a knee coil. T2* gradient echo and fat suppression sequences were used for coronal and sagittal planes images acquisition (Additional file 4). Two of the authors (JA, MR) blindly assessed complete MRI sequences and scored each RC-joint by consensus agreement. For each RC-joint, evaluators assigned a value from 0 (normal) to 4 (severely abnormal) for the parameters osteophyte formation, synovial effusion, osseous edema and subchondral bone damage (adapted from [34]).

### Gross anatomy and histopathology

Immediately after MRI, gross anatomy was evaluated by consensus agreement between four blinded evaluators (AV, JA, MP, FJV) by using an ordinal scale adapted to assess degenerative changes in the articular surface. Immediately after, synovium and cartilage samples were collected and split for histopathology and gene expression analyses. Synovium was harvested from the dorsal aspect of each joint. Slices of full-thickness cartilage were collected from the medial aspect of the radiocarpal bone and the distal radius and from areas of maximal injury. Samples were fixed in 10% formalin, embedded in paraffin, cut into 5 μm sections and stained with haematoxylin-eosin (H&E). Cartilage histochemistry was performed in additional sections stained by Safranin O-Fast Green (SOFG) to assess proteoglycan content by stain uptake [42, 43]. Three sections from each sample for both H&E and SOFG staining were assessed by one blinded external evaluator, who assigned scores following an ordinal scale (scales adapted from [42], Additional file 3.B). Parameters included in the scales were analyzed both separately and summed up to provide overall score. Histochemistry assessment by SOFG was separately evaluated.

### Gene expression

Samples destined to gene expression analysis were frozen and RNA was isolated with the Trizol (Qiagen)/Chloroform/Isopropanol method. The kit DNAseTurbo (Ambion) was used to remove genomic DNA and RNA quantity and purity were measured with the NanoDrop ND-1000 Spectrophotometer (Thermofisher). The Mean ± S.E.M of the ratio 260/280 was 1.935 ± 0.071 for cartilage and 1.945 ± 0.096 for synovium, reflecting appropriate RNA purity [44]. Subsequently, 1 μg of RNA was retrotranscripted into cDNA with the SuperScript-II System (Life Technologies), following the manufacturers’ instructions. The expression of genes related to ECM production, destruction/remodeling and joint inflammation were analyzed by RT-qPCR using the Fast SYBR Green Master Mix and the StepOne RT-qPCR System device (all from Applied Biosystems). Full list of genes and corresponding primers can be found in Additional file 5. The gene expression levels were determined by the ΔΔCt method using a normalization factor calculated as the geometric mean of two housekeeping genes (GAPDH and B2M). Methodology for gene expression analyses was performed as previously reported [45].

### Statistical analysis

Statistical analysis was performed with the SPSS 15.0 (SPSS, Inc.). Normality was tested with the Shapiro-Wilk test. The aim of using both carpi of each animal for a two-phase design was to compare them at different end-points but not along the study, since the procedure was the same in both phases up to two months. Therefore, the data collected along the study from both phase 1 and phase 2 carpi were added at each time-point until two months of evolution. Thus, for clinical, synovial and radiological parameters, the number of RC-joints assessed were *n* = 36 until two months (8 RC-joints control, 14 RC-joints MSC-naïve, 14 RC-joints MSC-primed), and *n* = 18 from two to six months (4 RC-joints control, 7 RC-joints MSC-naïve, 7 RC-joints MSC-primed). For clinical and synovial parameters, values at each time-point were compared to those at the moment of the MSC-naïve, MSC-primed or control injection (Week 2 or 5) with the Wilcoxon test to study the evolution within each group. Additionally, the percentages of change at each time-point compared to the Time 0 were calculated in each group and compared between groups by the Kruskall-Wallis test followed by Dunn’s posthoc test. For radiology scores, the same test was used to assess differences between groups at each time-point and differences between time-points within each group were studied by the Friedman test. For MRI, gross anatomy, histopathology, histochemistry and gene expression analyses, the results were first compared between groups at each end-point by Kruskall-Wallis test followed by Dunn’s posthoc analysis, and subsequently between both end-points within each group with the Wilcoxon test. Significance was set at *p* < 0.05 for all analysis.

## Results

### Clinical follow-up

No statistically significant differences were found between groups at baseline (Time 0, pre-lesion) for clinical parameters. All horses developed lameness with marked joint effusion and local heat within 6 h following amphotericin-B injection. Signs of joint inflammation peaked at 24-48 h and were thereafter progressively decreasing throughout the study. Despite the transient overload of phase 1 forelimbs due to the arthritis induction in the phase 2, clinical worsening of the phase 1 carpi (i.e. increased lameness or inflammatory signs) was not detected.

**Fig. 1:**
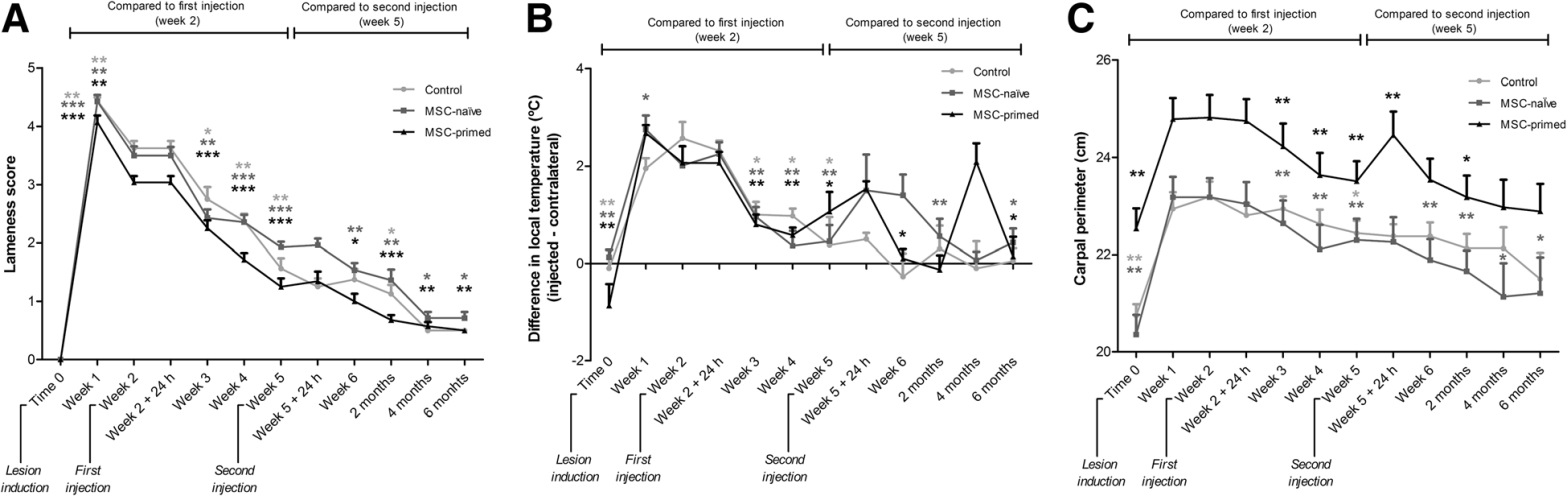
Results from clinical follow-up including lameness score, carpal heat and carpal perimeter. The two-phase study design aimed to perform paired comparison between two and six months’ end-points but not along the study because both phases 1 and phase 2 carpi underwent the same procedure. Thus, data collected from both phase 1 and phase 2 carpi was used for analyses until two months. Therefore, number of radio-carpal (RC)-joints assessed for clinical parameters were *n* = 36 until two months (8 RC-joints control, 14 RC-joints MSC-naïve, 14 RC-joints MSC-primed), and *n* = 18 from two to six months (4 RC-joints control, 7 RC-joints MSC-naïve, 7 RC-joints MSC-primed). Light grey lines and asterisks correspond to control group, dark grey lines and asterisks to MSC-naïve group and black lines and asterisks to MSC-primed group. Evolution of each parameter is shown as Mean ± SEM values for each group at each time-point. Significant differences are shown between time-points within each group: time-points from Time 0 (pre-lesion) to Week 5 (just before second injection) were compared to the time of the first injection (Week 2). Subsequent time-points were compared to second injection (Week 5). **a** Lameness score (0–5) along the study. **b** Carpal heat along the study is shown as the difference between the injected carpus and the contralateral one (injected – contralateral) at each time-point (°C). For the phase 2, it was checked that local temperature had returned to basal values in phase 1 carpi and that there were no differences between opposite carpi. **c** Carpal perimeter along the study is shown in centimeters. Note significant reduction of carpal perimeter in groups MSC-naïve and MSC-primed on Week 3 (one week after first injection) but not in control group until Week 5. Also note the significant increase in carpal perimeter 24 h after second injection only in MSC-primed group, which is subsequently normalized (* = *p* < 0.05, ** = *p* < 0.01, *** = *p* < 0.001)

Lameness and local heat did not show significant differences between groups but suggested faster decrease in both MSC-treated groups (Fig. 1a and b). After first treatment or control injection, carpal perimeter was significantly reduced (*p* < 0.01) in both MSC-treated groups sooner than in control (Fig. 1c). In addition, comparison between groups within each time-point was performed by using the percentage of change of the carpal perimeter over Time 0 (pre-lesion). MSC-primed group presented significantly lower percentage of change (i.e. less increase in the carpal perimeter) compared to control or to both control and MSC-naïve groups. This was observed at all time-points after both injections (*p* < 0.05), except at 24 h after both first (Week 2) and second (Week 5) injection (Additional file 6).

Second injection of allogeneic cells led to an increase in carpal heat (non-significant) and perimeter (*p <* 0.01) in MSC-primed group within next 24 h, but not in the MSC-naïve group. This effect was transient, not accompanied by increased lameness or signs of pain, and spontaneously resolved by one week later (Fig. 1b-c). Only one animal in the MSC-primed group developed marked effusion with signs of pain within few hours after the second injection in the phase 2, but the reaction was self-limiting and resolved with no anti-inflammatory medication (analgesia was provided as described above).

### Synovial fluid follow-up

**Fig. 2:**
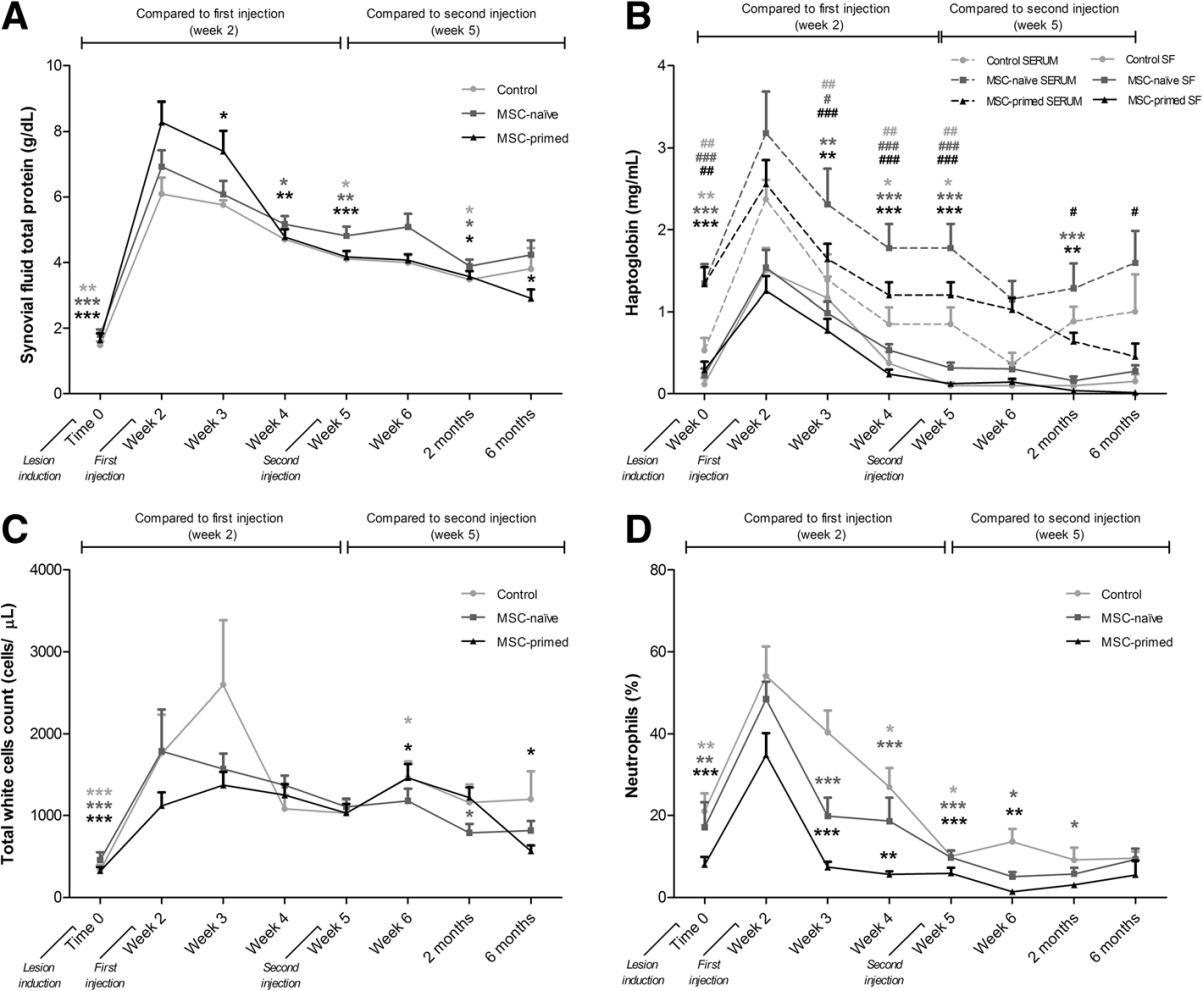
Synovial fluid (SF) assessment included total protein, haptoglobin, and total and differential white cells counts. The two-phase study design aimed to perform paired comparison between two and six months’ end-points but not along the study because both phase 1 and phase 2 carpi underwent the same procedure. Thus, data collected from phase 1 and phase 2 carpi was used for analyses until two months. Therefore, number of radio-carpal (RC)-joints assessed for synovial parameters were *n* = 36 until two months (8 RC-joints control, 14 RC-joints MSC-naïve, 14 RC-joints MSC-primed), and *n* = 18 for six months (4 RC-joints control, 7 RC-joints MSC-naïve, 7 RC-joints MSC-primed). Light grey lines and symbols represent control group, dark grey lines and symbols represent MSC-naïve group and black lines and symbols represent MSC-primed group. Results are presented as Mean ± SEM of each parameter at the evaluated time-points. Significant differences are shown between time-points within each group: time-points from Time 0 (pre-lesion) to Week 5 (just before second injection) were compared to the time of the first injection (Week 2). Subsequent time-points were compared to second injection (Week 5). **a** Evolution of the SF total protein (g/dl). **b** Evolution of the haptoglobin concentration (mg/ml) in SF and serum along the study. Continuous lines represent SF values and discontinuous lines represent serum values (control, *n* = 4 animals; MSC-naïve, *n* = 7 animals; MSC-primed, *n* = 7 animals). Asterisks (*) are used for significant differences in SF and hashes (#) are used for significant differences in serum. **c** Evolution of the SF total white cells count (cells/μL) along the study. **d** Evolution of the percentage (%) of neutrophils in SF along the study. (* = *p* < 0.05, ** = *p* < 0.01, *** = *p <* 0.001; # = *p <* 0.05, ## = *p <* 0.01, ### = *p <* 0.001)

No statistically significant differences were found between groups at baseline (Time 0, pre-lesion) for synovial parameters. The highest values for the synovial parameters assessed were found at Week 2 after the arthritis induction, just before the first injection of MSC-naïve, MSC-primed or control substance. Significant differences were not found between groups at each time-point for synovial parameters, but MSC-treated groups provided quicker normalization: one week after the first treatment or control injection (Week 3), TP was significantly reduced only in the MSC-primed group and one week later (Week 4) also by the MSC-naïve group, but not in the control group until Week 5. At the end of the study (six months), only MSC-primed group showed significantly reduced TP values (*p* < 0.05) compared to the moment of the second injection (Fig. 2a).

Similarly, haptoglobin was reduced in SF one week after first injection (Week 3) in both MSC-treated groups (*p* < 0.01) but not in the control (*p* < 0.05) until the next week (Week 4). Synovial haptoglobin was significantly reduced after second injection of MSC-naïve (*p* < 0.001) and MSC-primed (*p <* 0.01) at two months and only MSC-primed group showed significant haptoglobin reduction also in serum (*p <* 0.05) at two and at six months (Fig. 2b) (at the beginning of the phase 2 [four months], values of serum haptoglobin were returned to basal values in the three groups).

Total WCC peaked at Week 3 in control group, whereas at this time-point tWCC did not increase over Week 2 (first treatment injection) in the MSC-treated groups. However, tWCC increased in both control and MSC-primed groups (*p <* 0.05) one week after the second injection (Week 6), but the values were significantly reduced by the end of the study only in the MSC-primed group (*p* < 0.05) (Fig. 2c). Percentage of neutrophils was significantly reduced only by MSC-treated groups by one week after both first (Week 3, *p <* 0.001) and second injections (Week 6, *p <* 0.05 for MSC-naïve; *p <* 0.01 for MSC-primed) (Fig. 2d). Neither lymphocytes nor macrophages showed significant changes in their SF counts (data not shown).

### Imaging assessment

**Fig. 3:**
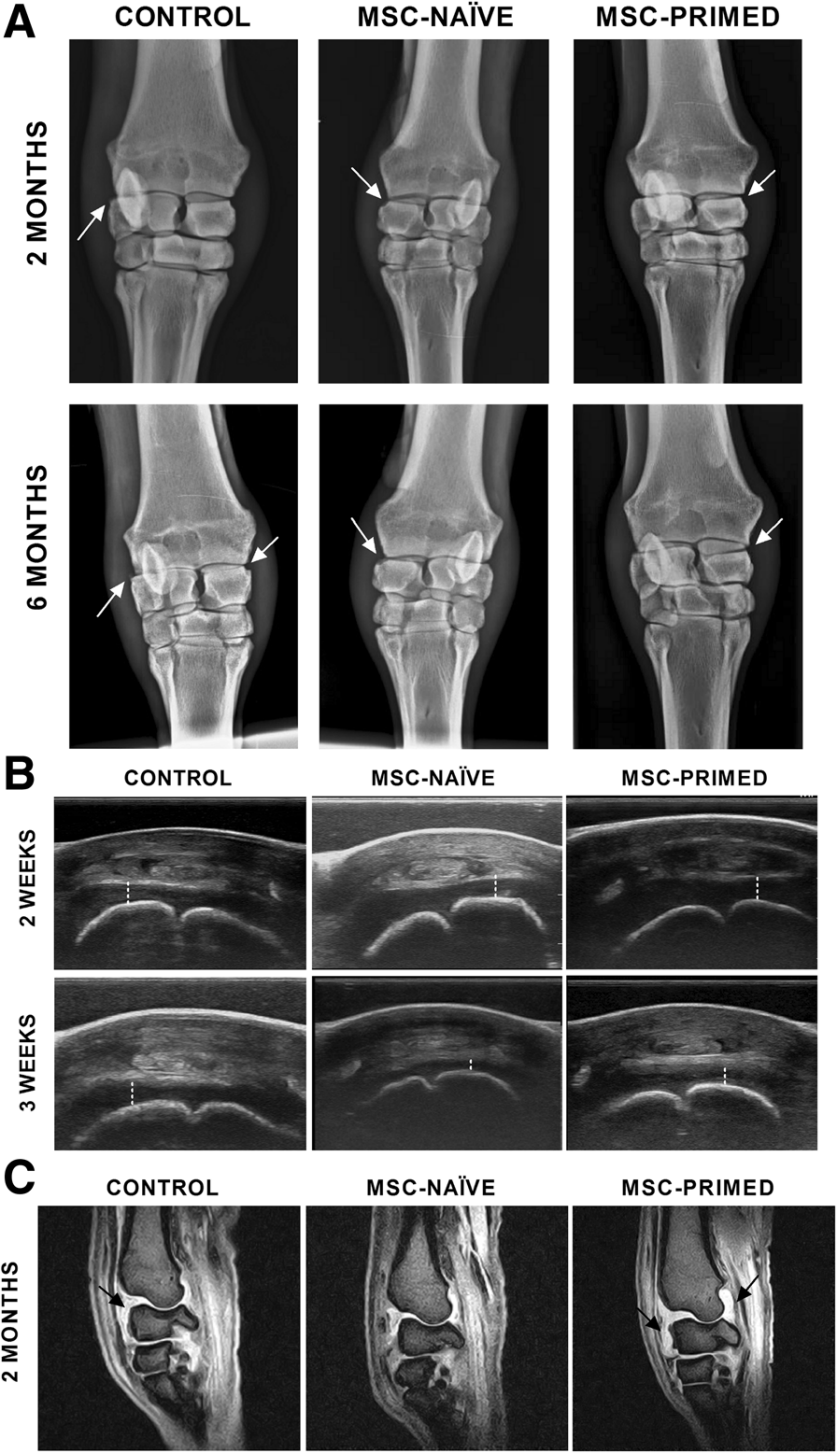
Representative images of the radiology, ultrasonography and magnetic resonance imaging (MRI) assessments, taken from one animal from each group (control, MSC-naïve and MSC-primed). **a** Radiologic images (frontal views) are presented for two (score 3) and six months (score 3–4) of progression. Score 6 is the highest possible. Joint space narrowing and marginal bone remodeling (white arrows) were progressively observed; note that the radiologic evolution was similar between the three groups. **b** Ultrasound images are presented for Week 2 (first injection) and Week 3. Note that synovial effusion (dashed line) was faster reduced in both MSC-naïve and MSC-primed groups than in control. **c** Magnetic resonance images (T2* gradient echo [GE], sagittal planes) are presented for showing the different synovial effusion (black arrows) found between groups at two months (scores 1, 0 and 3 were assigned to the images showed for control, MSC-naïve and MSC-primed groups, respectively)

Radiology showed similar progression in the three groups with significantly higher scores at all assessed time-points compared to Time 0 (pre-lesion), but without significant differences neither between subsequent time-points within each group, nor between groups at any time-point (Table 1, Fig. 3a). Ultrasonography showed decreased joint effusion and capsulitis one week after first injection of both MSC-naïve and MSC-primed treatments (Fig. 3b). Slight irregularities in the articular surfaces began to be detected by two months in the three groups and progressed along the study with final outcome similar between groups. In MRI, it was noticed lower synovial effusion in RC-joints where MSC-naïve were injected compared to RC-joints where MSC-primed were injected at two months (*p <* 0.05) and both MSC-treatments reduced subchondral bone damage compared to control (non-significant) (Table 2, Fig. 3c).

**Table 1:** Radiology scores (Mean ± SD) for each group (control, MSC-naïve, MSC-primed) at each assessed time-point

Time	Control	MSC-naïve	MSC-primed
Week 0 (pre-lesion)	0.00 ± 0.00^a^ *n = 8*	0.00 ± 0.00^a^ *n = 14*	0.00 ± 0.00^a^ *n = 14*
Week 2 (first injection)	2.50 ± 1.31^b^ *n = 8*	1.93 ± 0.99^b^ *n = 14*	1.43 ± 0.85^b^ *n = 14*
2 months	3.00 ± 0.93^b^ *n = 8*	3.43 ± 1.82^b^ *n = 14*	2.71 ± 1.33^b^ *n = 14*
4 months	2.50 ± 0.58^b^ *n = 4*	3.86 ± 1.77^b^ *n = 7*	2.71 ± 1.70^b^ *n = 7*
6 months	2.50 ± 1.00^b^ *C4*	4.43 ± 1.90^b^ *n = 7*	3.00 ± 1.29^b^ *n = 7*

The number (n) of radio-carpal joints assessed is showed for each time-point within each group. Higher scores mean higher severity of the radiologic findings, 6 being the highest possible score. Different letters mean statistically significant differences (*p <* 0.05). Note that significant differences were only found between pre-lesion and subsequent evaluations within each group, but not between groups at any time

**Table 2:** Magnetic resonance imaging (MRI) scores (Mean ± SD) for each group (control, MSC-naïve, MSC-primed)

MRI grading parameter	Two months end-point *(Phase 2 carpi)*			Six months end-point *(Phase 1 carpi)*		
	Control *n = 4*	MSC-naïve *n = 7*	MSC-primed *n = 7*	Control *n = 4*	MSC-naïve *n = 7*	MSC-primed *n = 7*
Osteophyte formation	0.25 ± 0.50	0.00 ± 0.00	0.00 ± 0.00	0.00 ± 0.00	0.33 ± 0.58	0.00 ± 0.00
Synovial effusion	1.50 ± 0.58^a,b^	0.67 ± 0.58^a,c^	2.25 ± 0.50^b^	1.25 ± 0.50	1.33 ± 0.58	1.75 ± 0.96
Osseous edema	0.25 ± 0.50	0.33 ± 0.58	1.25 ± 1.26	0.00 ± 0.00	0.00 ± 0.00	0.25 ± 0.50
Subchondral bone damage	1.00 ± 0.00	0.33 ± 0.58	0.50 ± 0.58	0.25 ± 0.50	0.33 ± 0.58	0.75 ± 0.50

Scores are shown for each MRI grading parameter in each group at each assessed end-point (six and two months, phases 1 and 2). The number (n) of radio-carpal (RC) joints assessed in each group at each end-point is showed in the table. Higher scores mean higher severity of the MRI findings, 4 being the highest possible score for each parameter. Different letters mean statistically significant differences (*p <* 0.05). Note that significant differences were only found between groups for the parameter synovial effusion at two months (phase 2), but not for other parameters or between two and six months

### Macroscopic and histopathological assessments

**Fig. 4:**
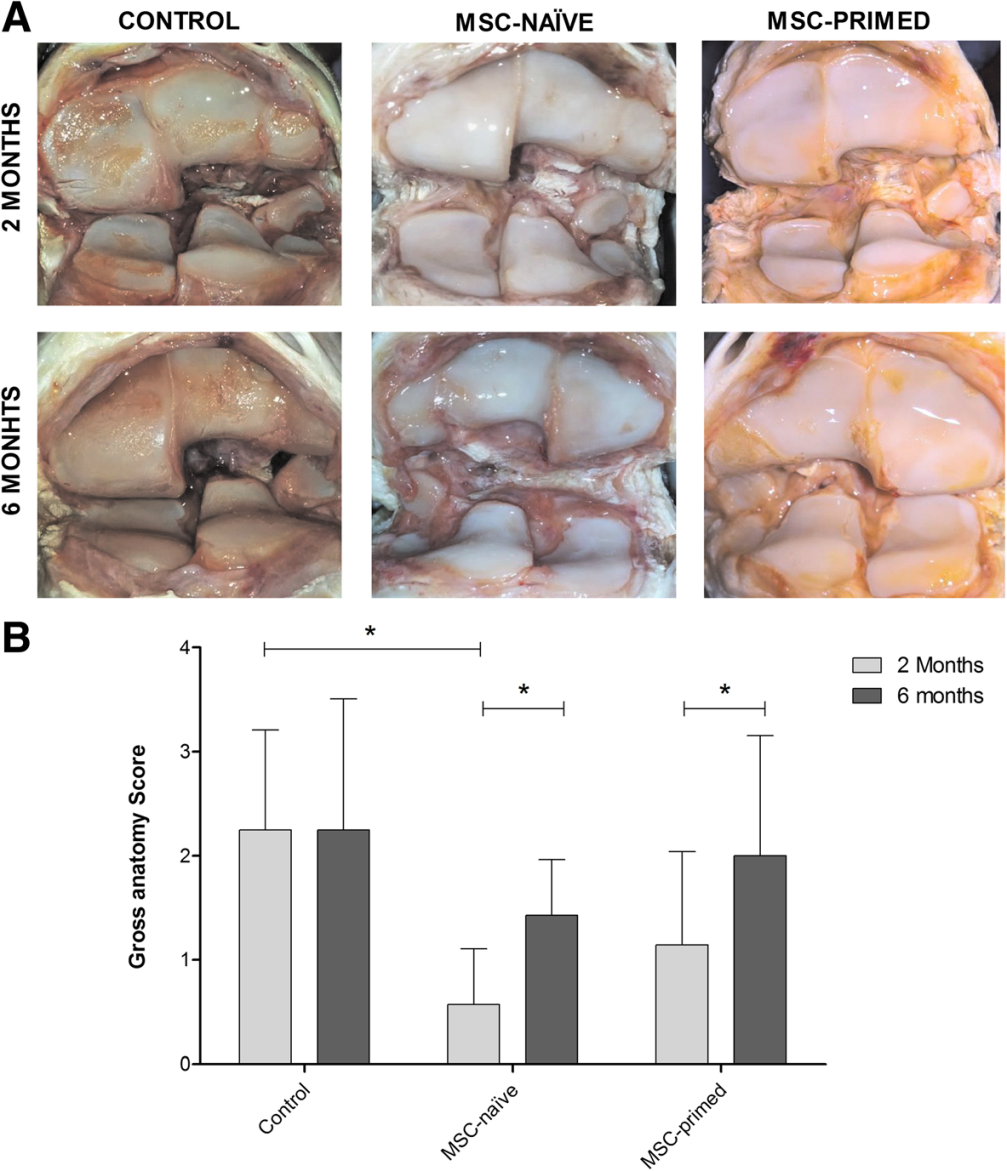
Gross anatomy outcome was assessed by visual examination of joint surfaces. **a** Representative images of the macroscopic appearance of the articular cartilage surface in one animal from each group (control, MSC-naïve, MSC-primed) at both end-points (two and six months). Assigned scores in these images were 3 (control, both 2 and 6 months), 0 (MSC-naïve, 2 months), 1 (MSC-naïve, 6 months; MSC-primed, 2 months) and 2 (MSC primed, 6 months). **b** Mean **±** SEM of the gross anatomy score for each group at two months end-point (phase 2 lesion, light grey bars; control, *n* = 4 radio-carpal [RC]-joints; MSC-naïve, *n* = 7 RC-joints; MSC-primed, *n* = 7 RC-joints) and at six months end-point (phase 1 lesion, dark grey bars; control, *n* = 4 RC-joints; MSC-naïve, *n* = 7 RC-joints; MSC-primed, *n* = 7 RC-joints). The highest possible score is 4. (* = *p* < 0.05)

Overall, macroscopic observation of the articular surfaces revealed lesions in the cartilage, from discoloration and fibrillation to erosions of different extent, which were suggestive of degenerative changes led by the inflammation induced. Gross anatomy scores were similar between the three groups at six months (phase 1 RC-joints), but lower at two months (phase 2 RC-joints) in both MSC-treated groups compared to control (MSC-naïve, *p* < 0.05; MSC-primed, non-significant). Within each group, both MSC-treated groups showed significantly lower scores at two months of progression compared to six months (*p* < 0.05), whereas the control group showed similar scores at both end-points (Fig. 4).

**Fig. 5:**
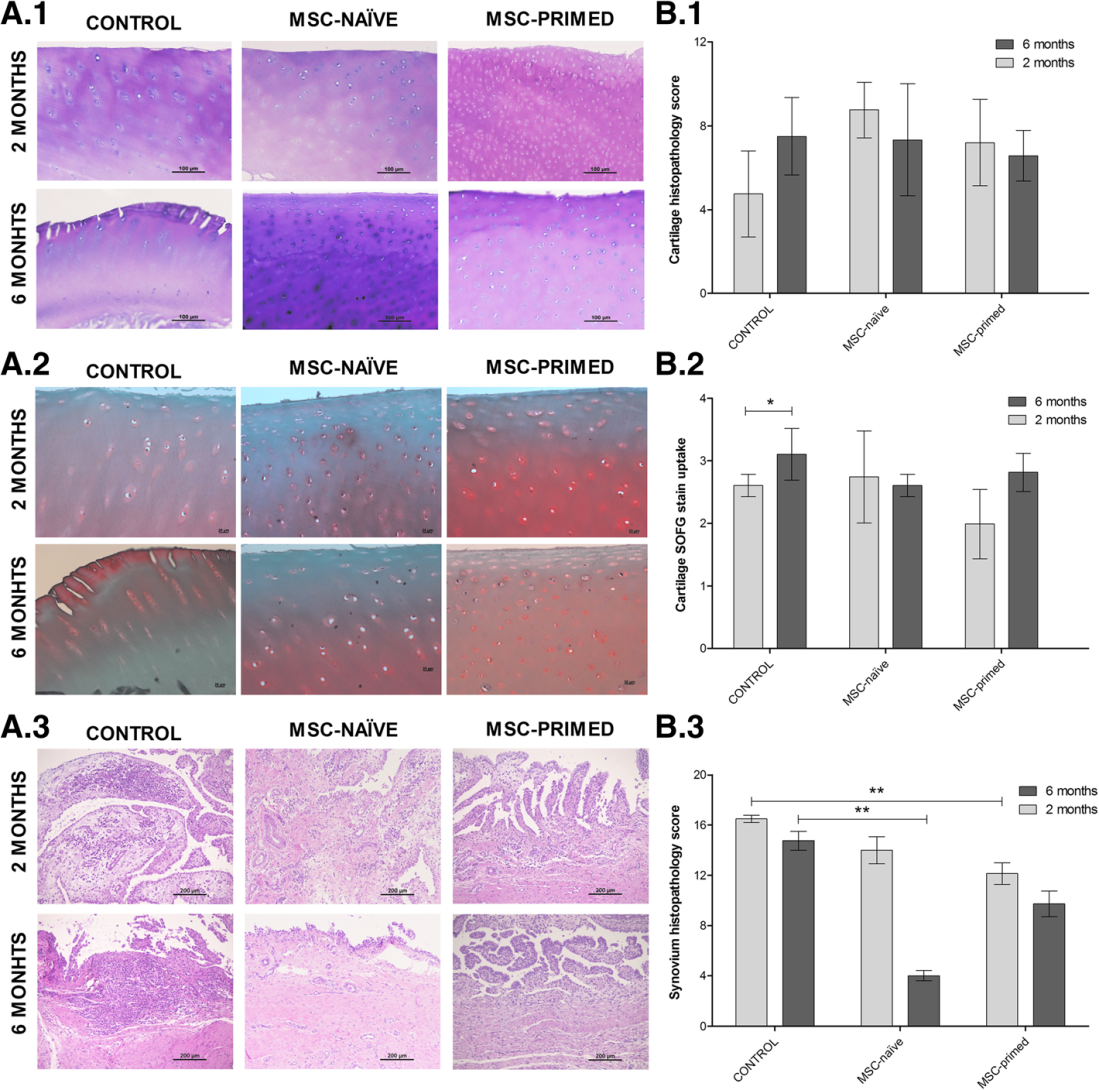
Histopathology and histochemical assessment of cartilage and synovium was performed assigning scores to several parameters*.* **a** Representative histologic images from one animal of each group (control, MSC-naïve, MSC-primed) at both end-points (two and six months); (**A.1**) Cartilage (hematoxylin-eosin [H&E], × 20): different degrees of fissuring/fibrillation of the cartilage surface, chondrocyte necrosis and areas of focal cell loss can be observed, with more severe abnormalities for control animal at six months. Overall scores assigned to each image are as follows: score 6 (control, 2 months), score 8 (control, 6 months; MSC-naïve, 2 and 6 months; MSC-primed, 2 months), score 7 (MSC-primed, 6 months); (**A.2**) Cartilage histochemistry (safranin O-fast green [SOFG], × 20): different degrees of SOFG stain uptake related to proteoglycan content. Scores assigned to each image are as follows: score 4 (control, 6 months), score 3 (control, 2 months; MSC-naïve, 2 and 6 months; MSC-primed, 6 months), score 2 (MSC-primed, 2 months); (**A.3**) Synovium (H&E, × 100): different degrees of mononuclear cells infiltration and changes in vascularity can be observed, more markedly in the control group at both end-points. Overall scores assigned to each representative image are as follows: score 16 (control, 2 months), score 14 (control, 6 months; MSC-naïve, 2 months), score 4 (MSC-naïve, 6 months), score 12 (MSC-primed, 2 months), score 10 (MSC-primed, 6 months). **b** Mean **±** SEM of the scores assigned to each group at two months (phase 2, light grey bars; control, *n* = 4 radio-carpal [RC]-joints; MSC-naïve, *n* = 7 RC-joints; MSC-primed, *n* = 7 RC-joints) and at six months (phase 1, dark grey bars; control, *n* = 4 RC-joints; MSC-naïve, *n* = 7 RC-joints; MSC-primed, *n* = 7 RC-joints); (**B.1**) in cartilage histopathology (H&E, total histopathologic score [sum of scores from each parameter], highest possible score is 12); (**B.2**) in cartilage histochemistry (SOFG, highest possible score is 4) and (**B.3**) in synovium histopathology (H&E, total histopathologic score [sum of scores from each parameter], highest possible score is 20) (* = *p* < 0.05; ** = *p* < 0.01)

On the whole, histopathology revealed findings related to damaged cartilage such as fibrillation and chondrocyte loss, and signs of synovium inflammation like cellular infiltration or subintimal changes. Overall score did not reveal differences between phase 1 and 2 within each group neither for cartilage nor for synovium. Comparison between groups at each phase neither showed significant differences in cartilage histopathology (Fig. 5a.1, b.1), nor in SOFG histochemistry, but control group showed significantly higher score (*p* < 0.05) at six months compared to two months, meaning reduced proteoglycan content at long-term, while neither MSC-naïve nor MSC-primed groups showed significant differences between phase 1 and 2 (Fig. 5a.2, b.2). On the other hand, synovium evaluation showed significantly lower total score in MSC-primed (*p* < 0.01) and MSC-naïve groups (*p <* 0.01), compared to control, at two (phase 2) and six months (phase 1), respectively (Fig. 5a.3, b.3). Analyzing each individual parameter, MSC-primed group showed lower scores at two months for vascularity (*p* < 0.05) and subintimal fibrosis (*p <* 0.01); whereas MSC-naïve group provided lower score at six months for cellular infiltration (*p* < 0.05), subintimal edema (*p <* 0.01) and subintimal fibrosis (*p <* 0.01) (Additional file 7).

### Gene expression analysis

#### Cartilage

**Fig. 6:**
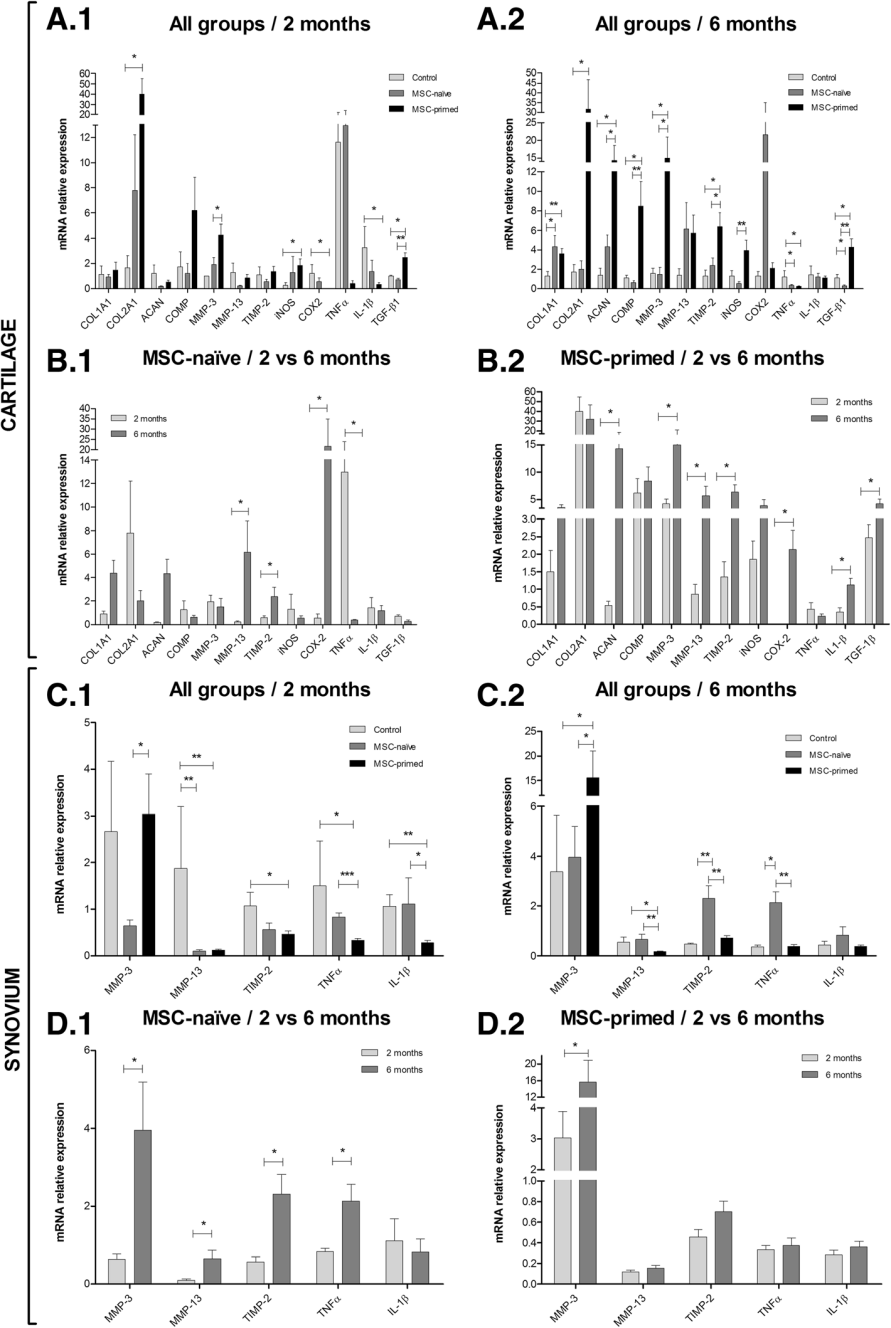
Gene expression related to extracellular matrix production, destruction/remodeling and joint inflammation in cartilage and synovium. Results are shown as Mean ± SEM of the relative mRNA expression. **a** Gene expression in cartilage compared between all the three groups (**A.1**) at two months end-point (phase 2 lesion; control, *n* = 4 radio-carpal [RC]-joints; MSC-naïve, *n* = 7 RC-joints; MSC-primed, *n* = 7 RC-joints) and (**A.2**) at six months end-point (phase 1 lesion; control, *n* = 4 RC-joints; MSC-naïve, *n* = 7 RC-joints; MSC-primed, *n* = 7 RC-joints). **b** Gene expression in cartilage compared between two and six months (**B.1**) for MSC-naïve group (*n* = 7 animals; *n* = 7 RC-joints for two months and *n* = 7 RC-joints for six months) and (**B.2**) for MSC-primed group (*n* = 7 animals; *n* = 7 RC-joints for two months and *n* = 7 RC-joints for six months). **c** Gene expression in synovium compared between all the three groups (**C.1**) at two months end-point (phase 2 lesion; control, *n* = 4 RC-joints; MSC-naïve, *n* = 7 RC-joints; MSC-primed, *n* = 7 RC-joints) and (**C.2**) at six months end-point (phase 1 lesion; control, *n* = 4 RC-joints; MSC-naïve, *n* = 7 RC-joints; MSC-primed, *n* = 7 RC-joints). **d** Gene expression in synovium compared between two and six months (**D.1**) for MSC-naïve group (*n* = 7 animals; *n* = 7 RC-joints for two months and *n* = 7 RC-joints for six months) and (**D.2**) for MSC-primed group (*n* = 7 animals; *n* = 7 RC-joints for two months and *n* = 7 RC-joints for six months). Note that the Y axe of some graphs has been divided into two or three segments for clearer representation of smaller bars. COL1A1, collagen type I; COL2A1, collagen type II; ACAN, aggrecan; COMP, cartilage oligomeric matrix protein; MMP-3, matrix metalloproteinase 3; MMP-13, matrix metalloproteinase 13; TIMP-2, tissue inhibitor of metalloproteinase 2; iNOS, inducible nitric oxide synthase; COX-2, cyclooxygenase 2; TNFα, tumor necrosis factor alpha; IL-1β, interleukin 1 beta; TGF-β1, transforming growth factor beta 1 (* = *p <* 0.05, ** = *p <* 0.01)

Cartilage from RC-joints from phase 2 (two months progression) of the MSC-primed group showed significant upregulation of collagen type II (COL2A1), inducible nitric oxide synthase (iNOS) and transforming growth factor (TGF)-β1 (*p <* 0.05) and downregulation of cyclooxygenase (COX)-2, interleukin (IL)-1β (*p* < 0.05) and TNFα (non-significant) compared to the control group. Additionally, MSC-primed group upregulated MMP-3 expression compared to MSC-naïve (Fig. 6a.1). At six months (RC-joints from phase 1), cartilage from both MSC-treated groups significantly upregulated collagen type I (COL1A1) (*p* < 0.05 MSC-naïve; *p* < 0.01 MSC-primed) and downregulated TNFα (*p <* 0.05) compared to the control. In addition, MSC-primed group showed significantly higher expression of COL2A1, aggrecan (ACAN), cartilage oligomeric matrix protein (COMP), MMP-3, tissue inhibitor of metalloproteinases (TIMP)-2 and TGF-β1 (*p* < 0.05) than control, and significant upregulation of iNOS (*p <* 0.01) compared to MSC-naïve (Fig. 6a.2). Comparison of cartilage gene expression between two and six months within each group did not show significant differences in the control group (data not shown). On the other hand, gene expression at six months compared to two months was significantly higher for MMP-13, TIMP-2 and COX2 (*p* < 0.05) in both MSC-treated groups; MSC-naïve group significantly downregulated TNFα (*p <* 0.05) and MSC-primed group showed higher expression of ACAN, MMP-3, IL-1β and TGF-β1 (*p <* 0.05) (Fig. 6b.1 and b.2).

#### Synovium

In synovium from RC-joints from phase 2 (two months progression), MMP-3 expression was significantly lower in MSC-naïve group (*p <* 0.05) than in MSC-primed, MMP-13 was significantly downregulated in both MSC-treated groups (*p <* 0.01) compared to control; and TIMP-2, TNFα (*p <* 0.05) and IL-1β (*p <* 0.01) were significantly downregulated in MSC-primed group compared to control (Fig. 6c.1). At six months (RC-joints from phase 1), significant MMP-3 upregulation and MMP-13 downregulation (*p <* 0.05) over control was found in MSC-primed group, and MSC-naïve treatment significantly upregulated TIMP-2 (*p <* 0.01) and TNFα (control, *p <* 0.05; MSC-primed, *p <* 0.01) compared to the other groups (Fig. 6c.2). When comparing synovium gene expression between two and six months within each group, differences were not found in control group (data not shown). Both MSC-treated groups showed significantly lower expression of MMP-3 at two than at six months (*p <* 0.05). In addition, significant upregulation of MMP-13, TIMP-2 and TNFα (*p <* 0.05) was found at six compared to two months in the MSC-naïve group (Fig. 6d.1 and d.2).

## Discussion

This study reports beneficial effects of repeated IA administration of allogeneic MSCs, both unstimulated and primed with proinflammatory cytokines, in an equine model of chemically induced-OA. Despite the lack of huge differences between the two types of MSC-treatments, the overall outcome suggested increased anti-inflammatory and regulatory abilities of MSC-primed. Beneficial effects were mainly found at limiting inflammation and subsequent cartilage degradation in an early stage. While long-term benefit of cellular therapies is desired, previous studies also showed that MSCs can improve the early healing response, but are limited for enhancing the long-term outcome [33, 35, 46]. This might be associated to MHC expression induction by proinflammatory cytokines [17], potentially leading to higher MSC-primed immunogenicity that at first could be dampened by their increased immunosuppressive properties but might compromise their long-term survival and effectivity [23].

To assess whether MSC-treatments may achieve different outcomes depending on the progression, and provided that previous studies found short-term benefit that was not maintained in the longer-term, the study was designed to enable two end-points evaluation. This two-phase study design aimed at reducing the number of animals by using both RC-joints of each one instead of duplicating animals. It is quite common to use both opposite joints in large animal models [34, 36], but some considerations should be taken into account. To begin with, as MSCs elicit paracrine signaling, it cannot be completely discarded remote influence in contralateral joints, even though MSC migration from one joint to another appears to be unlikely [31, 32]. Secondly, arthritis induction led to transient altered weight-bearing of the contralateral forelimb, but clinical affectation of the contralateral RC-joints was not detected in any case because of this transient overloading.

Although amphotericin-B model does not mimic the most common pathogenesis of OA (i.e. post-traumatic), it is acknowledged that inflammation plays a major role in initiation and progression of cartilage damage. Furthermore, this study mainly aimed at assessing regulatory abilities of MSCs in limiting inflammation and subsequent cartilage erosive lesions, thus relevant inflammatory component playing an important role. Even though results obtained using this model may not be directly extrapolated into naturally-occurring OA, the inflammatory regulation and the delay in cartilage degradation observed are of significance and can contribute to further clarify the role exerted by MSCs in OA.

The IA injection of amphotericin-B resulted in an arthritis model accordingly to previous reports [11, 47]. Previous studies in the horse using MSCs in experimental models showed no clinical differences between treated and control groups [19, 34], but other studies in naturally-occurring joint pathologies showed enhanced clinical outcome [28, 48]. In our study, differences in the clinical progress between control and MSC-treated groups were slight, but the evolution of clinical parameters suggested faster normalization in the MSC-treated groups. Accordingly, synovial parameters were also normalized faster in the MSC-treated RC-joints. In the SF, the most remarkable effect of the MSC-treatments was the significant reduction of the parameters earlier than in the control. Reduction of TP has also been reported by using autologous MSCs in the same model [11] and in our study TP was quicker reduced in the MSC-primed group. Haptoglobin has been recently described in the horse as a potential SF inflammatory marker in a study using the same lesion model [38]. In the present study, significant haptoglobin reduction in SF was first reached by both MSC-treatments. Similarly, MSC-treatments normalized the percentages of white cells populations sooner than control, even if the tWCC reduction was not significant as observed with autologous MSCs [19].

Therefore, taking together both clinical and synovial findings, it was noticed an anti-inflammatory effect elicited by both MSC-treatments at first stage after administration, especially after the first injection when joint inflammation was higher. Repeated administration of allogeneic MSC-naïve was well tolerated, accordingly to that reported in healthy joints [27]. However, second injection of allogeneic MSC-primed led to a slight transient inflammatory reaction, indicating that these cells might have increased immunogenicity as discussed above [17]. Interestingly, only second injection of these cells but not the first one produced this effect. This might have been due to the generation of immune memory, as it was recently reported in the horse the production of functional antibodies against MHC-mismatched MSCs [49]. One limitation of our study was that MHC-matching was not assessed between donors and receptors and authors hypothesize that alloantibodies production might have been implied, but the results of our study do not allow confirmation of this hypothesis. Nevertheless, this inflammatory reaction was transient and spontaneously resolved, but raises questions about the survival and immune allorecognition of MSC-primed requiring further investigation.

Regarding imaging assessment, ultrasonography suggested quicker reduction of the joint effusion shortly after first injection of both MSC-treatments, in agreement with clinical and synovial parameters. On the other hand, higher synovial effusion later noticed by MRI in MSC-primed group at two months would concord with the mild reaction observed after second injection of MSC-primed, but not for MSC-naïve, as discussed above. Beyond these observations, radiology and MRI were unable to reveal obvious differences between groups. Similarly, in a sheep model it was not observed radiologic changes but ultrasonography was useful for revealing joint alterations [33], and radiology and MRI did not provide different outcomes between control and MSC-treated joints in previous equine studies [34].

Improved cartilage macroscopic appearance was observed in both MSC-treated groups, which was more obvious at short-term thus suggesting a delaying effect on the progression of cartilage degradative changes. Accordingly, a previous study using the same lesion model showed enhanced macroscopic appearance after autologous MSC administration, but did not report differences between two and six months of progression [11]. On the other hand, cartilage histopathology did not show significant improvement in MSC-treated groups. Whereas gross appearance is overall assessed, histopathology evaluation is carried out on specific sites, which might explain the differential results observed between gross and histopathology scores. Previous studies using different equine OA models neither provided remarkable improvement after autologous MSC administration in the histopathology of cartilage and synovium [19, 35]. However, in this study synovium histopathology did reveal significantly improved score at two months (phase 2) for MSC-primed and at six months (phase 1) for MSC-naïve. These findings might suggest anti-inflammatory potential earlier elicited by MSC-primed that might not be reached by MSC-naïve until longer-term. As SOFG stains proteoglycans in the cartilage ECM, higher stain uptake reflects better quality of the repaired tissue [43]. Despite no statistical differences between control and treated groups, control group showed significantly higher loss of proteoglycans at six than at two months. However, MSC-treated groups did not show this significantly worsening evolution, thus suggesting that progression of cartilage degradation might have been delayed by both treatments, accordingly to that suggested by the macroscopic appearance. Moreover, even though not significant differences were found between groups, MSC-primed showed higher proteoglycan content at two months. Overall, histologic exam showed enhanced effects of MSC-primed at short-term which were not apparently maintained at longer-term. This observation might be also related with faster elimination of these cells, potentially related to the immunogenicity issues aforementioned [23].

Despite similar histological cartilage outcome between groups, the upregulation of COL2A1, ACAN and COMP in MSC-primed group in the phase 1 (six months) suggested an attempt of cartilage reparation with better quality of the healing tissue, as these genes are related with cartilage ECM production and they are downregulated during OA [50]. Similarly, increased collagen type II in MSC-treated joints has been reported in a rat model [51] and MSCs provided higher aggrecan content in an equine model [34]. Upregulation of COL1A1 in both MSC-treated groups might be also indicating an attempt of the chondrocytes to initiate repairs even if COL1A1 is related to fibroblastic rather than to chondrocytic phenotype [52].

Changes in MMPs and TIMP expression after both MSC-treatments were mainly found in the synovium, according to previous report of this structure as a major target of MSC therapy for modulating degrading enzymes [53]. Both MSC-treatments reduced MMP-13 expression in synovium at two months end-point (phase 2), whereas only MSC-primed also showed MMP-13 downregulation at six months (phase 1). Accordingly, MMP-13 expression is reduced in synovium explants cultured in the supernatant of MSCs primed with TNFα and IFNγ [54]. On the other hand, even if MMP-3 is a degradative enzyme, a protective role of the cartilage has also been proposed [55, 56], so its upregulation in both synovium and cartilage from MSC-primed group might be related to an attempt of restoring the functional balance in the joint. Furthermore, the MMP inhibitor TIMP-2 was found upregulated in cartilage from MSC-primed group at six months and its expression tended to change towards the same direction than MMP-13 in the synovium of this group, maybe aiming at reversing the imbalance between them present during OA [57].

Although nitric oxide (NO) is considered an OA pro-inflammatory marker, protective roles in the joint [58] and immunomodulatory properties have also been attributed to NO [59]. Thus, the role of iNOS upregulation in cartilage from MSC-primed group might not be detrimental but regulatory [60]. Expression of iNOS by chondrocytes is mainly induced by IL-1 and TNFα, which are the major pro-inflammatory cytokines involved in OA and they are in vitro upregulated in equine chondrocytes after inflammatory stimuli [61] and in vivo in cartilage from equine OA carpi [62]. Expression of TNFα was reduced in the cartilage of both MSC-treated groups at six months, similarly to that previously reported in vitro [54]. However, only MSC-primed also downregulated TNFα at two months in both the cartilage, even though not significantly, and the synovium. Furthermore, whereas MSC-naïve did not significantly change the expression of IL-1β over control in any case, MSC-primed significantly downregulated IL-1β at two months in cartilage and at six months in synovium. Therefore, higher anti-inflammatory effect of MSC-primed on joint tissues was suggested. The different end-points at which each change was found to be significant may have been due to different cytokine dynamics during OA [63]. TNFα and IL-1β are able to induce the secretion of PGE2, synthesized by COX2, which increased concentrations and gene expression are related with decreased proteoglycans synthesis and increased ECM destruction during OA [14, 64, 65]. PGE2 decreased in equine cartilage explants exposed to MSC-primed conditioned media [54] and in SF from OA experimental horses treated with autologous MSCs [19].We observed lower expression of COX2 at two than at six months in cartilage from both MSC-treated groups, but only MSC-primed significantly downregulated COX2 compared to control. This finding might be related with higher proteoglycan content, as shown by SOFG cartilage staining, in the MSC-primed group at two months compared to the other conditions. TGF-β1 is considered to have a cartilage protective role through stimulating ECM components synthesis and by reducing MMP expression [66], as well as regulating the joint inflammation [67]. Thus, the upregulation of TGF-β1 in cartilage after MSC-primed at both end-points suggested enhanced repair stimulation and cartilage protection [66]. This growth factor predominates during the remission phase of the OA [63], which might explain the higher expression observed at six months.

## Conclusions

Overall, MSC-primed treatment suggested more powerful anti-inflammatory and regulatory effects, but the slight inflammatory reaction observed after their second injection requires further investigation about immunogenicity implications. Beneficial effects were mostly observed at short-term, which might be due to MSC short-term life after in vivo administration, especially for the allogeneic MHC-mismatched ones. Nevertheless, our results suggest an in vivo enhanced regulatory ability of MSC-primed for limiting inflammation and subsequent cartilage degradation in this model of joint pathology. While further investigation is warranted to clarify the immunogenic implications of using allogeneic MSC-primed and the extent to which these results can be extrapolated, findings of this study contribute to better understand the mechanisms underlying the beneficial effects of MSCs in joint pathology and how MSC therapeutic potential could be influenced.

## Additional files

Additional file 1: Schematic representation of the study design. (DOCX 62 kb)

Additional file 2: Characterization of equine bone marrow derived mesenchymal stem cells. (PDF 102 kb)

Additional file 3: Scoring systems used for radiologic, gross anatomy and histopathologic assessments. (DOCX 24 kb)

Additional file 4: Magnetic resonance imaging parameters set for each sequence. (DOCX 14 kb)

Additional file 5: Primers used for gene expression by real time quantitative polymerase chain reaction. (DOCX 17 kb)

Additional file 6: Percentage of change of the carpal perimeter at each time-point compared to Time 0 (pre-lesion). (DOCX 49 kb)

Additional file 7: Histopathologic assessment of the synovium presented by separate parameters. (DOCX 117 kb)

## Abbreviations

ACAN: Aggrecan
BM-MSCs: Bone marrow derived mesenchymal stem cells
COL1A1: Collagen type I
COL2A1: Collagen type II
COMP: Cartilage oligomeric matrix protein
COX2: Cyclooxygenase 2
DMEM: Dulbecco’s Modified Eagle’s Medium
DMSO: Dimethyl-sulfoxide
dWCC: Differential white cells count
ECM: Extracellular matrix
FBS: Fetal bovine serum
H&E: Hematoxylin-eosin
HR: Heart rate
IA: Intra-articular
IFNγ: Interferon gamma
IL: Interleukine
iNOS: Inducible nitric oxide synthase
LRS: Lactate’s Ringer Solution
MHC: Major histocompatibility complex
MMP: Matrix metalloproteinase
MRI: Magnetic resonance imaging
MSC: Mesenchymal stem cell
OA: Osteoarthritis
RC: Radio-carpal
RR: Respiratory rate
RT-qPCR: Real time quantitative polymerase chain reaction
SF: Synovial fluid
SOFG: Safranin O-fast green
TGF-β1: Transforming growth factor beta 1
TIMP: Tissue inhibitor of metalloproteinase
TNFα: Tumor necrosis factor alpha
TP: Total protein
tWCC: Total white cells count
