# Supplementary material for: Assessment of effectiveness and safety of repeat administration of proinflammatory primed allogeneic mesenchymal stem cells in an equine model of chemically induced osteoarthritis
Source: BMC Vet Res. 2018 Aug 17;14:241. doi: 10.1186/s12917-018-1556-3 (PMC6098603; doi:10.1186/s12917-018-1556-3)
Supplement: Supplementary file 1 — Schematic representation of the study design. (DOCX 62 kb) [file 12917_2018_1556_MOESM1_ESM.docx]

**Supplementary material 1.** *Schematic representation of the study design.*


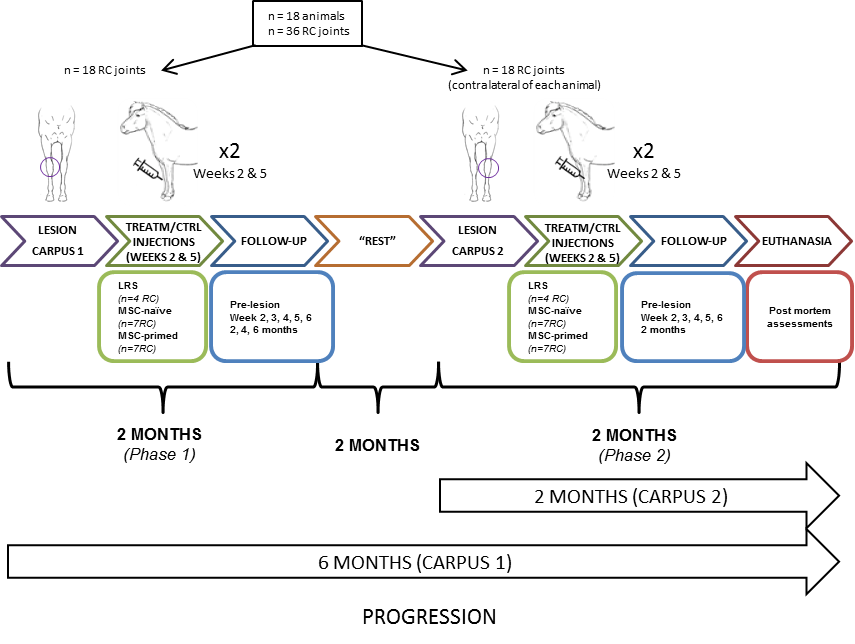


In phase 1, lesion was created in one random radio-carpal (RC)-joint of each animal. On weeks 2 and 5 after inducing the arthritis, repeat injections of corresponding substance (TREATM = treatment: 10·10^6^ unstimulated mesenchymal stem cells [MSC-naïve] or 10·10^6^ of TNFα + IFNγ-stimulated cells [MSC-primed]; or CTRL = control: Lactate’s Ringer Solution [LRS]) were administered to corresponding RC-joints from animals in the MSC-naïve, MSC-primed and control groups; respectively. Serial follow-up (clinical, synovial and imaging assessments) was carried out up to two months and animals “rested” for additional two months. Thus, four months after inducing the first lesion, phase 2 was carried out in which the contralateral RC-joint of each animal was subjected to the same corresponding procedure: second lesion was induced, identical injections administered –depending on the group- and follow-up performed during two months. At this point (six months since the first lesion induction for phase 1, two months since the second one for phase 2), animals were euthanized to conduct post-mortem assessments including magnetic resonance imaging (MRI), gross anatomy, histopathology and gene expression analysis. Therefore, first lesion induced in phase 1 had progressed along six months (long-term) while second lesion created in phase 2 had progressed for only two months (short-term), providing two different end-points for post-mortem assessments without duplicating the number of animals involved.
