# Supplementary material for: Assessment of effectiveness and safety of repeat administration of proinflammatory primed allogeneic mesenchymal stem cells in an equine model of chemically induced osteoarthritis
Source: BMC Vet Res. 2018 Aug 17;14:241. doi: 10.1186/s12917-018-1556-3 (PMC6098603; doi:10.1186/s12917-018-1556-3)

Supplementary material 2.- *Characterization of equine bone marrow derived mesenchymal stem cells (BM-MSCs).*

After their isolation, cells were characterized at passage 3 by the expression of defined markers and their ability to differentiate into adipocytes, osteoblasts and chondrocytes. Expression of positive markers CD90, CD105 and CD44 was assessed by both real time quantitative polymerase reaction (RT-qPCR) and flow cytometry. Lack of expression of the negative markers CD34 and CD45 was evaluated by RT-qPCR. Methodology for performing RT-qPCR and analyzing gene expression results was carried out as described in the manuscript. Primers used for characterization are detailed below:

Flow cytometry for assessing surface expression of markers was carried out as previously described

GENE	*Accession number*	*Primer sequence (5'–3')*	*Amplicon size (bp)*
		House-keeping	
GAPDH	NM_001163856	F:GGCAAGTTCCATGGCACAGT<br>R:CACAACATATTCAGCACCAGCAT	128
B2M	NM_001082502.2	F: TCGTCCTGCTCGGGCTACT<br>R: ATTCTCTGCTGGGTGACGTGA	102
		Characterization cell surface markers	
CD90	EU881920	F:TGCGAACTCCGCCTCTCT<br>R:GCTTATGCCCTCGCACTTG	93
CD105	XM_001500078	F:GACGGAAAATGTGGTCAGTAATGA<br>R:GCGAGAGGCTCTCCGTGTT	100
CD44	NM 001085435	F: CCCACGGATCTGAAACAAGTG 95<br>R: TTCTGGAATTTGAGGTCTCCGTAT	95
CD45	AY_114350	F:TGATTCCCAGAAATGACCATGTA<br>R:ACATTTTGGGCTTGTCCTGTAAC	100
CD34	XM_001491596	F:CACTAAACCCTCTACATCATTTTCTCCTA<br>R:GGCAGATACCTTGAGTCAATTTCA	150

(Ranera et al., 2011). Briefly, the cells were suspended in PBS/2 mM EDTA at 10^6cells/ml. 50 µl aliquots of cells were transferred to FACS tubes and incubated for 15 min at 4ºC with mouse anti-human monoclonal antibodies CD90-PE (BDPharmingen), CD105-FITC (R&D Systems) and CD44-FITC (Abcam). Anti-horse reactivity of antibodies was previously tested (Ranera et al., 2011; Barrachina et al., 2016). Subsequently, cells were washed with PBS (Gibco), diluted in 500 µl of PBS/2 mM EDTA and analyzed with the fluorescence-activated cell sorter FACSARIA (BD Biosciences). Gating strategy was performed as described in Barrachina et al., (2016) and data were analyzed with FACSDIVA 5.0.1 software.

The induction of differentiation into the osteogenic, adipogenic and chondrogenic lineages was performed by using the specific media described in Barrachina et al., (2017) and successful differentiation was checked by the specific staining Alizarin Red, Oil-O Red and Alcian Blue as previously described (Barrachina et al., 2017).

In the following figure, data obtained from characterization assays are presented. Panel A shows Mean ± S.E.M (n=4) of the mRNA relative expression of CD90, CD105, CD44, CD34 and CD45.

Panel B presents Mean ± S.E.M (n=4) of the percentage of positive cells for the surface markers CD90, CD105 and CD44 determined by flow cytometry. Panel C shows the results from the differentiation assays: negative control (C.1), lipid droplets stained by Oil-O Red in the adipogenesis (C.2), calcium deposits stained by Alizarin Red in the cells undergoing osteogenesis (C.3) and proteoglycan rich extracellular matrix is stained by Alcian Blue after chondrogenic induction in 3D (micro-mass) culture (C.4).

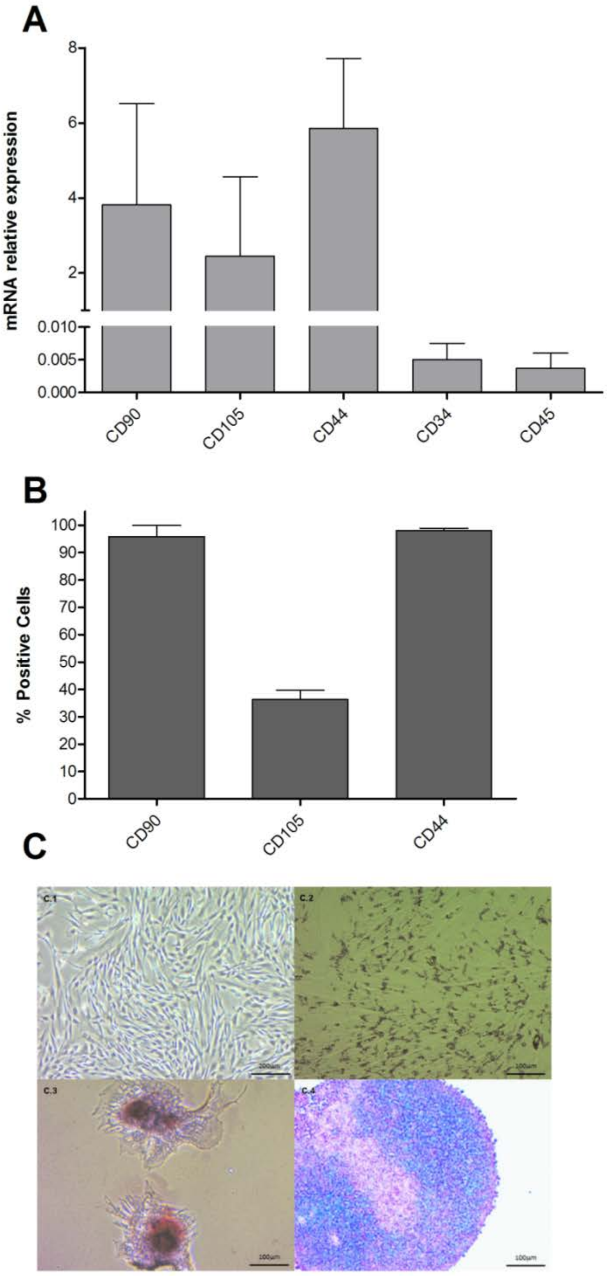

Supplement: Supplementary file 2 — Characterization of equine bone marrow derived mesenchymal stem cells. (PDF 102 kb) [file 12917_2018_1556_MOESM2_ESM.pdf]
