# Supplementary material for: Assessment of effectiveness and safety of repeat administration of proinflammatory primed allogeneic mesenchymal stem cells in an equine model of chemically induced osteoarthritis
Source: BMC Vet Res. 2018 Aug 17;14:241. doi: 10.1186/s12917-018-1556-3 (PMC6098603; doi:10.1186/s12917-018-1556-3)
Supplement: Supplementary file 3 — Scoring systems used for radiologic, gross anatomy and histopathologic assessments. (DOCX 24 kb) [file 12917_2018_1556_MOESM3_ESM.docx]

**Supplementary material 3**

**A)** Scoring system used for radiologic images assessment, based on a subjective ordinal scale adapted from ([Mokbel et al., 2011](#_ENREF_50)).

| Score | Description |
| --- | --- |
| 0 | Normal |
| 1 | Slight joint space narrowing  and/or  Slight bone remodeling in articular margins (no osteolysis) |
| 2 | Mild joint space narrowing  and  Mild bone remodeling in articular margins (no osteolysis) |
| 3 | Moderate joint space narrowing  and  Mild bone remodeling in articular margins (no osteolysis) |
| 4 | Moderate joint space narrowing  and/or  Moderate bone remodeling in articular margins (with/without osteolysis) |
| 5 | Severe joint space narrowing  and  Moderate bone remodeling in articular margins (with/without osteolysis) |
| 6 | Severe joint space narrowing  and  Severe bone remodeling in articular margins (with osteolysis) |

**B)** Gross anatomy and histopathology scoring systems based on a subjective ordinal scale adapted from the OARSI recommendations ([McIlwraith et al., 2010](#_ENREF_48)).

| Gross anatomy scoring | | | |
| --- | --- | --- | --- |
| Score | Description | | |
| 0 | Normal | | |
| 1 | Focal and superficial small size lesions, presenting one or several of the following findings:  - Discoloration  - Swelling/fibrillation  - Very superficial erosion | | |
| 2 | Presence of only one of the following findings:  - Extensive superficial lesions as described in score 1 (discoloration, swelling/fibrillation)  - Focal partial-thickness erosion | | |
| 3 | Simultaneous presence of the findings decribed in score 2:  - Extensive superficial lesions as described in score 1 (discoloration, swelling/fibrillation)  - Focal partial-thickness erosion | | |
| 4 | Extensive partial-thickness erosion and/or full-thickness erosion | | |
|  | | | |
| Cartilage histopathology scoring | | | |
| Chondrocyte necrosis | | 0 | Normal section without necrosis |
|  |  | 1 | No more than one necrotic cell located near the articular surface per 20x objective |
|  |  | 2 | 1-2 necrotic cells located near the articular surface per 20x objective |
|  |  | 3 | 2-3 necrotic cells located near the articular surface per 20x objective |
|  |  | 4 | 3-4 necrotic cells located near the articular surface per 20x objective |
| Fibrillation /fissuring | | 0 | No fibrillation/fissuring of the articular cartilage surface |
|  |  | 1 | Fibrillation/fissuring of the articular cartilage restricted to surface and superficial zone |
|  |  | 2 | Fissuring that extends into the middle zone |
|  |  | 3 | Fissuring that extends to the level of the deep zone |
|  |  | 4 | Fissuring that extends into the deep zone |
| Focal cell loss | | 0 | Normal cell population throughout the section |
|  |  | 1 | 10-20% area of acellularity per 20x field |
|  |  | 2 | 20-30% area of acellularity per 20x field |
|  |  | 3 | 40-50% area of acellularity per 20x field |
|  |  | 4 | >50% area of acellularity per 20x field |
| Cartilage histochemistry scoring | | | |
| Safranin O stain uptake | | 0 | Normal staining |
|  |  | 1 | < 25% loss of staining characteristics |
|  |  | 2 | 25-50% loss of staining characteristics |
|  |  | 3 | 50-75% loss of staining characteristics |
|  |  | 4 | > 75% loss of staining characteristics |

| Synovium histopathology scoring | | |
| --- | --- | --- |
| Cellular infiltration | 0 | No mononuclear cells in the section |
|  | 1 | Occasional small areas of mononuclear cells throughout the section |
|  | 2 | Mild presence of mononuclear cells in 25% of the section |
|  | 3 | Moderate presence of mononuclear cells in 25-50% of the section |
|  | 4 | Marked presence of mononuclear cells in >50% of the section |
| Vascularity | 0 | Normal |
|  | 1 | Slight increase in vessels in focal locations throughout the section |
|  | 2 | Mild increase in number and dilatation of vessels in focal locations throughout the section |
|  | 3 | Moderate increase in number and dilatation of vessels in up to 50% of the section |
|  | 4 | Marked increase in number and dilatation of vessels in >50% of the section |
| Intimal hyperplasia | 0 | None |
|  | 1 | Villi with 2-4 rows of intimal cells within the section |
|  | 2 | Villi with 4-5 rows of intimal cells over 25-50% of the section |
|  | 3 | Villi with 4-5 rows of intimal cells over 50% of the section |
|  | 4 | Villi with 5 or greater rows of intimal cells over 50% of the section |
| Subintimal edema | 0 | No edema |
|  | 1 | Slight edema detected within section |
|  | 2 | Mild edema within 25% of the section |
|  | 3 | Moderate edema within 25-50% of the section |
|  | 4 | Marked edema in >50% of the section |
| Subintimal fibrosis | 0 | Normal |
|  | 1 | Slight increase in fibrosis within the section |
|  | 2 | Mild increase in fibrosis in 25% of the section |
|  | 3 | Moderate increase in fibrosis in 25-50% of the section |
|  | 4 | Marked increase in fibrosis in >50% of the section |
