# Supplementary material for: Assessment of effectiveness and safety of repeat administration of proinflammatory primed allogeneic mesenchymal stem cells in an equine model of chemically induced osteoarthritis
Source: BMC Vet Res. 2018 Aug 17;14:241. doi: 10.1186/s12917-018-1556-3 (PMC6098603; doi:10.1186/s12917-018-1556-3)
Supplement: Supplementary file 4 — Magnetic resonance imaging parameters set for each sequence. (DOCX 14 kb) [file 12917_2018_1556_MOESM4_ESM.docx]

| *Sequence* | *Plane* | *TE (ms)* | *TR (ms)* | *FA (°)* | *Slice thickness (mm)* | *Slice Gap (mm)* |
| --- | --- | --- | --- | --- | --- | --- |
| T2* GE | Sagittal | 13.0 | 420.0 | 30° | 3.0 | 0.5 |
| T2* FS  Echo 1 | Coronal | 11.4 | 430.0 | 30° | 3.5 | 1.0 |
| T2* FS  Echo 2 | Coronal | 22.8 | 430.0 | 30° | 3.5 | 1.0 |

**Supplementary material 4.-** Magnetic resonance imaging (MRI) parameters set for each sequence. TE, echo time; TR, repetition time; FA, flip angle; FS, fat suppression; GE, gradient echo.
