# Supplementary material for: Assessment of effectiveness and safety of repeat administration of proinflammatory primed allogeneic mesenchymal stem cells in an equine model of chemically induced osteoarthritis
Source: BMC Vet Res. 2018 Aug 17;14:241. doi: 10.1186/s12917-018-1556-3 (PMC6098603; doi:10.1186/s12917-018-1556-3)
Supplement: Supplementary file 5 — Primers used for gene expression by real time quantitative polymerase chain reaction. (DOCX 17 kb) [file 12917_2018_1556_MOESM5_ESM.docx]

### Supplementary material 5.- Primers used for gene expression by RT-qPCR. GenBank accession numbers of the sequences used for primers design. Primers (F: Forward and R: Reverse) and length of the amplicon in base pair (bp). Genes were grouped in agreement with the functions and implications of encoded molecules. GAPDH, [Glyceraldehyde 3-phosphate dehydrogenase](https://en.wikipedia.org/wiki/Glyceraldehyde_3-phosphate_dehydrogenase); B2M, beta-2 microglobulin; COL1A1, collagen type I; COL2A1, collagen type II; ACAN, aggrecan; COMP, cartilage oligomeric matrix protein; MMP-3, matrix metalloproteinase 3; MMP-13, matrix metalloproteinase 13; TIMP-2, tissue inhibitor of metalloproteinase 2; iNOS, inducible nitric oxide synthase; COX-2, cyclooxygenase 2; TNFα, tumor necrosis factor alpha; IL-1β, interleukin 1 beta; TGF-β1, transforming growth factor beta 1.

| *Gene* | *Accesion number* | *Primer sequence (5‘–3’)* | *Amplicon size (bp)* |
| --- | --- | --- | --- |
| House-keeping | | | |
| GAPDH | NM_001163856 | F:GGCAAGTTCCATGGCACAGT  R:CACAACATATTCAGCACCAGCAT | 128 |
| B2M | NM_001082502.2 | F: TCGTCCTGCTCGGGCTACT  R: ATTCTCTGCTGGGTGACGTGA | 102 |
| Genes related with articular cartilage ECM production | | | |
| COL1A1 | AF034691 | F: ACACAGAGGTTTCAGTGGTTTGG  R: CACCATGGCTACCAGGTTCAC | 89 |
| COL2A1 | XM_005611082.1 | F: TTAGACGCCATGAAGGTTTTCTG  R: CTCTTGCTGCTCCACCAGTTCT | 101 |
| ACAN | AF019756 | F: CTACGACGCCATCTGCTACA  R: ACCGTCTGGATGGTGATGTC | 96 |
| COMP | AF325902 | F: GGCGACGCGCAAATAGA  R: GCCATTGAAGGCCGTGTAA | 111 |
| Genes related with articular cartilage ECM degradation/remodeling | | | |
| MMP-3 | NM_001082495 | F: TGATGTGACTGGCATTCAATCC  R: ATCGCACATGGCTAGTGTTCCA | 112 |
| MMP-13 | NM_001081804 | F: CCGAACCCTAAACATCCCAAA  R: GATGTAGGCGCCAGAAGAATCT | 121 |
| TIMP-2 | XM_005597879.1 | F: GAATGCAAGATCACGCGCT  R: TGCCCGTTGATGTTCTTCTCT | 101 |
| Inflammatory mediators and growth factors | | | |
| iNOS | AY027883 | F: CCAACAATGGCAACATCAGGT  R: TGAGCATTCCAGATCCGGA | 85 |
| COX-2 | AB041771 | F: GTTTGCATTTTTTGCCCAGC  R: ACTTAAATCCACCCCGTGACC | 103 |
| TNFα | EU438779 | F: CATGTTGTAGCAAACCCCCAA  R: TACAGCCCATCCAATGGTACC | 125 |
| IL-1β | NM_001082526.1 | F: CCCACCCTACAGCTGGAGAC  R: TCCACGTTGCCCTTGATTTC | 101 |
| TGFβ-1 | AF175709 | F: GTCCTTTGATGTCACCGGAGT  R: TGGAACTGAACCCGTT | 137 |
