# Supplementary material for: Assessment of effectiveness and safety of repeat administration of proinflammatory primed allogeneic mesenchymal stem cells in an equine model of chemically induced osteoarthritis
Source: BMC Vet Res. 2018 Aug 17;14:241. doi: 10.1186/s12917-018-1556-3 (PMC6098603; doi:10.1186/s12917-018-1556-3)
Supplement: Supplementary file 6 — Percentage of change of the carpal perimeter at each time-point compared to Time 0 (pre-lesion). (DOCX 49 kb) [file 12917_2018_1556_MOESM6_ESM.docx]

**Supplementary material 6.-** Percentage (%) of change of the carpal perimeter at each time-point compared to Time 0 (pre-lesion) was calculated for each carpus as [(T_0_ – T_n_) / T_0_] x 100; T_0_ being the value (carpal perimeter in centimeters) pre-lesion (Time 0) used as a reference and T_n_ being the value at each time-point. Results are shown as Mean ± SEM of the percentages of change for each group (until two months: control n=8 radio-carpal [RC]-joints, MSC-naïve n=14 RC-joints, MSC-primed n =14 RC-joints; from two to six months: control n=4 RC-joints, MSC-naïve n=7 RC-joints, MSC-primed n=7 RC-joints). Light grey bar represents control group, grey bar represents MSC-naïve group and black bar represents MSC-primed group. Significant differences between groups at each time point are presented (* = p<0.05, ** = p<0.01). MSC-primed group showed significantly lower percentage of change compared to the other groups at all time-points after treatment except at 24h after both injections (Weeks 2 and 5).
