# Supplementary material for: Assessment of effectiveness and safety of repeat administration of proinflammatory primed allogeneic mesenchymal stem cells in an equine model of chemically induced osteoarthritis
Source: BMC Vet Res. 2018 Aug 17;14:241. doi: 10.1186/s12917-018-1556-3 (PMC6098603; doi:10.1186/s12917-018-1556-3)
Supplement: Supplementary file 7 — Histopathologic assessment of the synovium presented by separate parameters. (DOCX 117 kb) [file 12917_2018_1556_MOESM7_ESM.docx]

**Supplementary material 7.** Mean **±** SEM score assigned to each separate parameter in the histologic synovium evaluation of the three groups (control, MSC-naïve and MSC-primed) at both end-points (two and six months). In each phase (phase 1, 6 months; phase 2, 2 months), synovium was obtained from: control, n=4 radio-carpal joints; MSC-naïve, n=7 radio-carpal joints; MSC-primed, n=7 radio-carpal joints). Light and dark grey bars indicate two and six months end-point, respectively. Scores were assigned ranging from 0 to 4, where higher score means higher severity, for the parameters showed in the Supplementary material 2.B: cellular infiltration, vascularization, intimal hyperplasia, subintimal edema and subintimal edema (*= p<0.05; ** = p<0.01).
